# Novel Experimental and Clinical Therapeutic Uses of Low-Molecular-Weight Heparin/Protamine Microparticles

**DOI:** 10.3390/pharmaceutics4010042

**Published:** 2012-01-11

**Authors:** Satoko Kishimoto, Masayuki Ishihara, Megumi Takikawa, Yasutaka Mori, Hidemi Hattori, Masanori Fujita, Shingo Nakamura

**Affiliations:** 1 Research Institute, National Defense Medical College, 3-2 Namiki, Tokorozawa, Saitama 359-8513, Japan; Email: resbiom@ndmc.ac.jp (S.K.); resbiom4@ndmc.ac.jp (Y.M.); h2@ndmc.ac.jp (H.H.); fujitama@ndmc.ac.jp (M.F.); 2 Research Fellow of the Japan Society for the Promotion of Science, Tokyo 102-8472, Japan; 3 Department of Plastic Surgery, National Defense Medical College, 3-2 Namiki, Tokorozawa, Saitama, 359-8513 Japan; Email: dr22057@ndmc.ac.jp; 4 Aeromedical Laboratory, Japan Air Self-Defense Force, 2-3 Inariyama, Sayama, Saitama 350-1394, Japan; 5 Department of Surgery, National Defense Medical College, 3-2 Namiki, Tokorozawa, Saitama 359-8513, Japan; Email: snaka@ndmc.ac.jp

**Keywords:** entrapment, microparticles, coating, polymeric drug delivery systems

## Abstract

Low-molecular-weight heparin/protamine microparticles (LMW-H/P MPs) were produced as a carrier for heparin-binding growth factors (GFs) and for various adhesive cells. A mixture of low-molecular-weight heparin (MW: approximately 5000 Da, 6.4 mg/mL) and protamine (MW: approximately 3000 Da, 10 mg/mL) at a ratio of 7:3 (vol:vol) yields a dispersion of microparticles (0.5–3 µm in diameter). LMW-H/P MPs immobilize, control the release and protect the activity of GFs. LMW-H/P MPs can also bind to cell surfaces, causing these cells to interact with the LMW-H/P MPs, inducing cells/MPs-aggregate formation and substantially promoting cellular viability. Furthermore, LMW-H/P MPs can efficiently bind to tissue culture plates and retain the binding of important GFs, such as fibroblast growth factor (FGF)-2. The LMW-H/P MPs-coated matrix with various GFs or cytokines may provide novel biomaterials that can control cellular activity such as growth and differentiation. Thus, LMW-H/P MPs are an excellent carrier for GFs and various cells and are an efficient coating matrix for cell cultures.

## 1. Introduction

Polyelectrolyte complexes (PECs) are generated by electrostatic interactions between oppositely charged polyelectrolytes. When this interaction occurs at non-equivalent ratios, nonstoichiometric PECs are produced, causing each PEC particle to carry an excess charge [1,2]. Proteins interact with both synthetic and natural PECs [3,4]. These binding characteristics, along with a simple preparation, allow PECs to be an excellent model for studying the *in vivo* behavior of charged biopolymers as well as having potential applications in medicine and biotechnology [5,6]. Reported data indicate that polyanions and polycations can bind to proteins below and above their isoelectric points, respectively. These interactions can result in soluble complexes, complex coacervation and/or the formation of amorphous precipitates [3,4,6]. Main aspects studied by different authors are compositions of PECs obtained under various experimental conditions, such as the strength and position of ionic sites, charge density, and rigidity of polymer chains as well as chemical properties such as solubility, pH, temperatureand concentration [1,5,6].

Electrostatic interactions are also important because of their similarity to biological interactions [7]. Interactions between proteins and nucleic acids, for example, play a role in the transcription process [3]. DNA/chitosan PECs [8], chitosan/chondroitin sulfate PECs and chitosan/hyaluronate PECs [9] function as gene and drug carriers. Moreover, PECs that are insoluble also have potential applications as membranes, microcapsules, micro/nano-particles and scaffolds for tissue engineering [10].

Particulate drug delivery systems have become widely employed in both experimental therapeutics and in the clinical setting to address a range of applications and disease states. Their popularity can be attributed to ease of application as a suspension and ease of manufacture. The size of the particles plays a substantial role in determining the properties of the final product and its potential applications. When injected into tissue, large particles (microparticles: about 1–5 µm in diameter) tend to stay where placed, while smaller particles (nanoparticles: about 50–200 nm in diameter) will circulate for a period of time determined by size, surface chemistry and other factors [11]. In general, microparticles will be useful if the particles are delivered locally. Examples include local anesthetic microparticles and microparticles including growth factor, antibiotics, and chemotherapeutics [12]. Heparinoids specifically interact with a variety of functional proteins with high affinity, including heparin-binding growth factors (GFs), cytokines, extracellular matrix components, and adhesion molecules [13,14,15]. Thus, heparin may be useful as a therapeutic agent in various pathological conditions that involve functional proteins; however, high-dose heparin cannot be used because of the excessive risk of bleeding [16]. In contrast, low-molecular-weight heparin (LMW-H, MW: approximately 5000 Da) has pharmacological and practical advantages compared with heparin. The lower protein binding activity of LMW-H produces a low, stable and predictable anticoagulant response, thereby bypassing the need for laboratory monitoring of drug levels to adjust the dosage [16]. In addition, one or two subcutaneous injections per day are sufficient to maintain therapeutic concentrations because of its longer plasma half-life [16].

On the other hand, protamine, a purified mixture of proteins obtained from fish sperm, neutralizes heparin and LMW-H by forming a stable complex that lacks anticoagulant activity [17]. Protamine is also in clinical use to reverse the anticoagulant activity of heparin following cardiopulmonary bypass as well as in cases of heparin-induced bleeding [18]. Furthermore, protamine is used as a carrier for insulin (protamine Hagedorn (NPH) insulin) [19]. 

**Figure 1:**
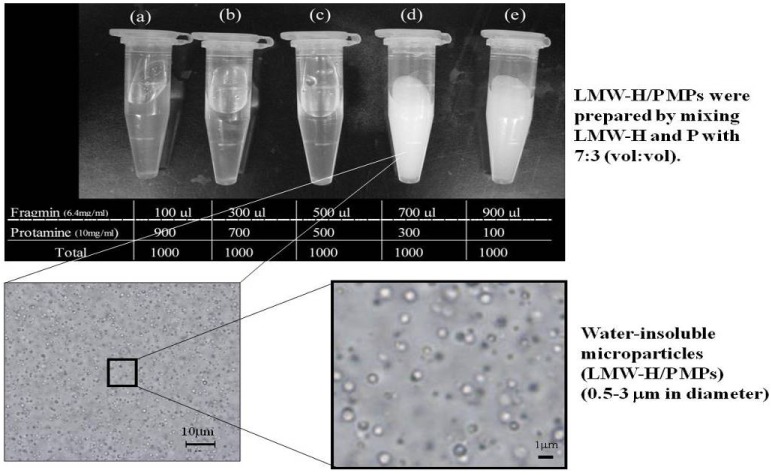
Preparation of Fragmin (LMW-H)/Protamine (P) MPs.

We previously prepared water-insoluble particles (>10 µm in diameter) by mixing non-anticoagulant heparin with chitosan. We then mixed fucoidan with chitosan and investigated the ability of the resulting insoluble fucoidan/chitosan microparticles to protect fibroblast growth factor (FGF)-2 activity [20,21]. We also prepared water-insoluble microparticles (0.5–3 µm in diameter) by mixing LMW-H (6.4 mg/mL) with protamine (10 mg/mL) at a ratio of 7:3 (vol:vol), and reported the ability of the resulting injectable low-molecular-weight heparin/protamine microparticles (LMW-H/P MPs) to protect FGF-2 activity (Figure 1) [22]. Furthermore, GFs from platelets in platelet-rich plasma (PRP) that were also involved in cell proliferation, migration, and angiogenesis were able to adsorb onto LMW-H/P MPs [23]. In another study, we used diluted LMW-H (≤0.32 mg/mL) as an anion molecule and the diluted protamine as a cation molecule to synthesize LMW-H/protamine nanoparticles (LMW-H/P NPs; approximately 120 nm in diameter) [24]. 

We prepared injectable microparticles (0.5–3 µm in diameter) by mixing LMW-H with protamine at a ratio of 7:3 (vol:vol).

We also reported that LMW-H/P MPs also bind to adipose-derived stromal cell (ASC) surfaces through specific interactions between LMW-H/P MPs and cell surface heparin-binding proteins such as some integrins. The interaction of the cells with LMW-H/P MPs resulted in cells/LMW-H/P MPs-aggregate formation. These aggregates substantially promoted cellular viability *in vitro*. Injection of the aggregates induced vascularization and fibrous tissue formation *in vivo* [25]. LMW-H/P MPs also bound to the surface of tumor cells (e.g., Lewis lung cancer cells (3LL), B16 melanoma cells (B16), and a human hepatoma cell line (Huh7)), promoting the aggregation of the tumor cells with the LMW-H/P MPs and increasing cell-to-cell interactions. The tumor cells/LMW-H/P MPs-aggregates substantially promoted tumor cell viability and proliferation *in vitro*, and reliably induced rapid tumor formation and tumor growth *in vivo* [26].

As a coating matrix, LMW-H/P MPs were efficiently bound to tissue culture plates. With the ability of LMW-H/P MPs to retain GFs, these microparticles could be very useful in cell culture. Human microvascular endothelial cells and human dermal fibroblast cells adhered to LMW-H/P MPs-coated tissue culture plates [27] and grew optimally in low fetal bovine serum (FBS) (1–2%) medium supplemented with FGF-2 (5 ng/mL). This protocol could make it possible to use low autologous serum (1–2%) for the culturing of human bone marrow-derived mesenchymal stem cells (BMSCs) and ASCs [28]. Furthermore, CD34+ hematopoietic progenitor cells (CD34+ cells) derived from mouse bone marrow exhibited a comparatively higher proliferation on LMW-H/P MPs-coated plates in hematopoietic progenitor growth medium (HPGM) supplemented with appropriate cytokines than those on uncoated plates [29]. In this review article, we describe the biocompatibility, bioactivity, and possible medical applications for LMW-H/P MPs.

## 2. Protein-Delivery Microparticles

### 2.1. Preparation of FGF-2-Containing LMW-H/P MPs and Their Applications

**Figure 2:**
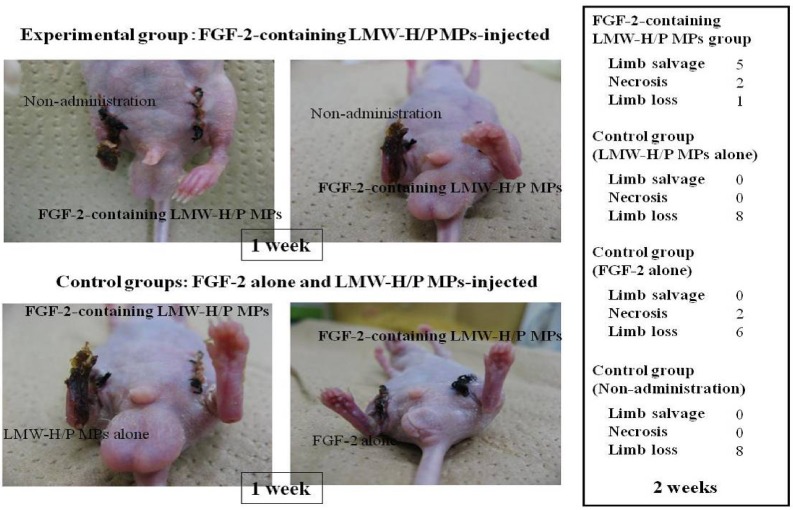
Prevention of limb loss in hindlimb Ischemic model by FGF-2-containing LMW-H/P MPs.

FGF-2 binds heparin and heparin-like molecules (heparinoids) with high affinity (Kd of 8.6 × 10^−9^ M). Some heparinoids can prolong the biological half-life of FGF-2 as well as protect FGF-2 from heat, acid, and proteolytic inactivation [30]. Similarly, the LMW-H/P MPs have high affinity for FGF-2 (Kd = 2.4 × 10^−9^ M) [22], and this interaction of FGF-2 with the LMW-H/P MPs can substantially prolong the biological half-life of FGF-2. The protection of FGF-2 against heat inactivation and trypsin degradation by the LMW-H/P MPs was effective in a concentration-dependent manner [22]. The results demonstrated that FGF-2 molecules are bound and stabilized on the LMW-H/P MPs, and that the FGF-2 molecules incorporated into the LMW-H/P MPs will be gradually released upon biodegradation of the microparticles* in vivo*. When the FGF-2-containing LMW-H/P MPs were subcutaneously injected into the backs of mice, neovascularization was induced near the injection site after 3 days. Neovascularization induced by the FGF-2-containing LMW-H/P MPs reached a maximum at 1 week, after which a slight decrease in the neovascularization rate occurred. No significant vascularization was observed after either the injection of FGF-2 or the LMW-H/P MPs alone [22]. Another study demonstrated advanced fat survival and capillary formation in FGF-2-containing LMW-H/P MPs-assist subdivided free fat-grafting groups in rats [31]. Furthermore, FGF-2-containing LMW-H/P MPs can stimulate local angiogenesis and arteriogenesis in animal models of hindlimb ischemia (Figure 2). Since all components used in the FGF-2-containing LMW-H/P MPs are also used clinically, we feel safety in a clinical setting is probable [32].

FGF-2-containing LMW-H/P MPs can stimulate local angiogenesis and arteriogenesis in animal models of hindlimb ischemia.

### 2.2. Preparation of PRP-Containing LMW-H/P MPs and Their Applications

PRP contains a high concentration of thrombocytes. In the α-granules of platelets, various GFs and other bioactive proteins augment tissue repair and regeneration processes [23,33,34]. Platelets contain more than 20 GFs, including platelet-derived growth factors (PDGFs), FGFs, hepatocyte growth factor (HGF), transforming growth factors (TGFs), and vascular endothelial growth factors (VEGFs), almost all of which are known to bind to heparin with high affinity. Recent studies suggest that GFs in PRP not only influence the viability of transferred cells but may also play bioactive roles in the regulation of proliferation and differentiation in adipocyte precursor cells. Clinical studies also documented the efficacy and safety of PRP in the stimulation and/or enhancement of native repair and regeneration processes during hard and soft tissue augmentation [33,34]. 

**Figure 3:**
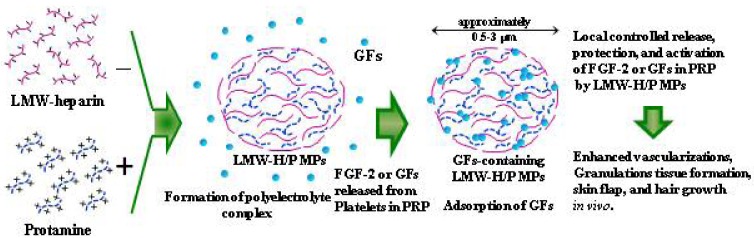
Mechanism on formation and function of GFs-containing LMW-H/P MPs.

The GFs in PRP are stably bound to LMW-H/P MPs *in vivo*. The GFs adsorbed onto LMW-H/P MPs may be gradually diffused and released upon biodegradation of LMW-H/P MPs. The effects of PRP-containing LMW-H/P MPs have been demonstrated in neovascularization and formation of granulation tissue using enhanced filtration of inflammatory cells in nude mice [23]. When PRP-containing LMW-H/P MPs were subcutaneously injected into the backs of mice, significantly higher neovascularization and granulation tissue formations with enhanced filtration of inflammatory cells were observed compared with the mouse group injected with PRP alone [23] (Figure 3). Compared to either PRP or LMW-H/P MPs alone, locally administered PRP-containing LMW-H/P MPs augmented the wound bed and substantially increased viability of rat dorsal paired pedicle skin flaps [35]. The improved flap survival was noted if PRP-containing LMW-H/P MPs was administered 2 days before the flap elevation [35]. PRP-containing LMW-H/P MPs may thus represent a promising new biomaterial for improving skin flaps, particularly in the field of reconstructive surgery.

**Figure 4:**
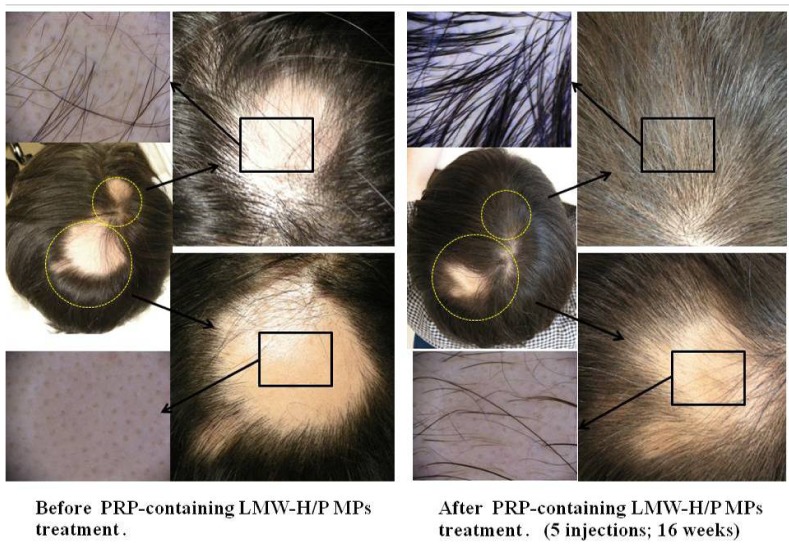
PRP-containing LMW-H/P MPs treatment for alopecia areata.

Clinical research was performed using autologous PRP-containing LMW-H/P MPs and PRP alone in 26 patients with thin hair (including 10 women) [36]. Hair growth and thickening following administration of both PRP-containing LMW-H/P MPs and PRP alone was observed in all patients compared with the control, but PRP-containing LMW-H/P MPs appeared to provide the most substantial change (Figure 4) [36]. Because of the use of autologous materials, this method using PRP-containing LMW-H/P MPs is simpler, cheaper, and has little side effect compared to conventional methods.

FGF-2 or GFs released from platelets in PRP are adsorbed onto polyelectrolyte complexes formed by mixing LMW-H with protamine (LMW-H/P MPs). The adsorbed GFs are locally released, protected, and activated by LMW-H/P MPs.

Compared with the control group, hair growth and thickening was observed in all patients following administration of both PRP-containing LMW-H/P MPs and PRP alone. However, PRP-containing LMW-H/P MPs appeared to provide the most substantial change.

## 3. Cell-Delivery Microparticles

### 3.1. LMW-H/P MPs as Stromal Cell Carriers

**Figure 5:**
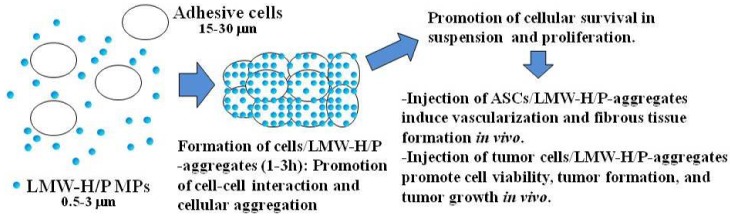
Mechanism on formation of cells/LMW-H/P MPs-aggregates as cell carrier.

LMW-H/P MPs as cell carriers can enhance cell viability as well as control the release of GFs. LMW-H/P MPs could substantially enhance the cellular viability of various suspension cultures, including human microvascular endothelial cells (hMVECs), human dermal fibroblasts (hDFCs) and ASCs [24]. In particular, ASCs have the potential to differentiate into skin, bone, cartilage, fat, myocardium, skeletal muscle and neurons [37]. Several reports indicate that the transplantation of human ASCs-cultured constructs significantly stimulates angiogenesis, wound repair, and re-epithelialization in athymic mice when compared with the corresponding human fibroblast-cultured constructs [38]. ASCs can be easily harvested with lower donor site morbidity compared with other pluripotent stem cell sources. Furthermore, ASCs can easily attach and proliferate in culture, and therefore, are available on a large scale even for autologous grafting in small animals such as rodents [39]. However, an application of ASCs for therapeutic angiogenesis and vasculogenesis requires microcarriers to act as injectable vehicles necessary for transplantation of ASCs. It was observed that LMW-H/P MPs could bind to the surface of the cells as mentioned above. The interaction of these cells with LMW-H/P MPs induced ASCs/LMW-H/P MPs-aggregate formation, and substantially promoted cell viability for at least 3 days in cell suspensions (Figure 5). The ASCs/LMW-H/P MPs-aggregates adhered and grew on suspension culture plates, and the aggregates similarly grew on type I collagen-coated plates. Furthermore, cultured ASCs secreted a significant amount of angiogenic GFs such as FGF-2, HGF, PDGF, and VEGF. These secreted GFs could have been retained within the ASCs/LMW-H/P MPs-aggregates. When the ASC/LMW-H/P MPs-aggregates were subcutaneously injected into the back of nude mice, a significant increase in neovascularization and fibrous tissue formation was observed near the injected site from 3 days to 2 weeks [24]. Taken together, these data indicate that ASCs/LMW-H/P MPs-aggregates are a useful and convenient biomaterial for angiogenesis and wound repair cellular therapy.

The interaction of adhesive cells with LMW-H/P MPs induced ASCs/LMW-H/P MPs-aggregate formation, and substantially promoted cell viability in cell suspensions *in vitro,* and injections of the aggregates significantly enhanced vascularization and fibrous tissue formations *in vivo.*

### 3.2. LMW-H/P MPs as Tumor Cell Carriers

Tumor cell transplantation models offer great strategy for cancer research. They include allograft transplantation and xenograft transplantation models. Allograft mouse tumor systems, also known as a syngeneic model, consist of tumor tissues derived from the same genetic background as a given mouse strain. The xenograft transplantation method involves actual human cancer cells or solid tumors which are transplanted into a host mouse [40]. Therefore, to effectively utilize tumor cell transplantation, the host mouse must possess an impaired immune system similar to nude mice, thereby allowing foreign tumor cells to survive and not be rejected by the host. In both cases of tumor cell transplantation, effective tumor cell carrier systems are required to improve cellular viability and growth as well as decreasing tumor cell rejection by the animals [40]. 

**Figure 6:**
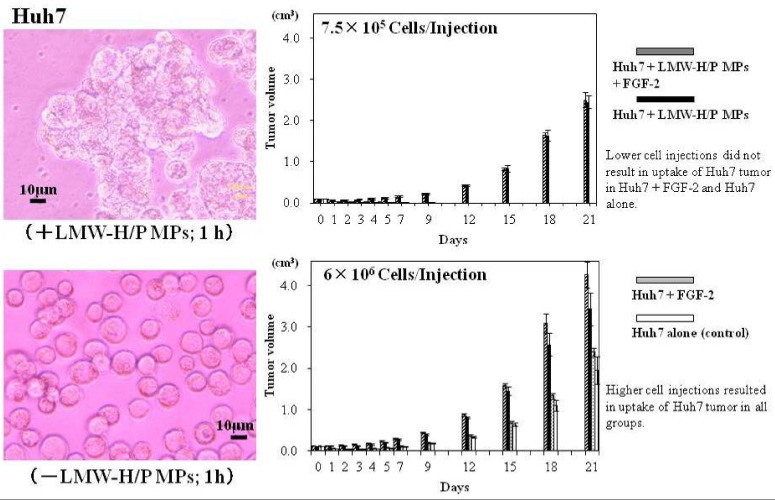
Huh7/LMW-H/P MPs aggregates and tumor formation and growth in nude mice.

The LMW-H/P MPs also bind to the surface of tumor cells (e.g., 3LL, B16, and Huh7), promote cell-to-cell interaction, and increase the aggregation of the tumor cells with the LMW/P MPs. These tumor cells/LMW-H/P MPs-aggregates substantially promote cell survival and proliferation of the tumor cells *in vitro* as well as reliably induce tumor formation and rapid tumor growth *in vivo* (Figure 6) [25]. Taken together, these data indicate that LMW-H/P MPs constitute a new, convenient and effective biomaterial that functions as a tumor cell carrier *in vivo*. The application of LMW-H/P MPs as a tumor cell carrier offers a more reliable model in both allograft and xenograft transplantation for cancer research [25]. 

LMW-H/P MPs bind to the surface of Huh7 cells, promote cell-to-cell interaction, and increase the aggregation of the tumor cells with the LMW/P MPs. These tumor cells/LMW-H/P MPs-aggregates substantially promote cell survival and proliferation of the tumor cells *in vitro* as well as reliably induce tumor formation and rapid tumor growth *in vivo*.

## 4. A Cell Culture System Using LMW-H/P MPs-Coated Plates

### 4.1. Various Types of Cell Cultures Using LMW-H/P MPs-Coated Plates

**Figure 7:**
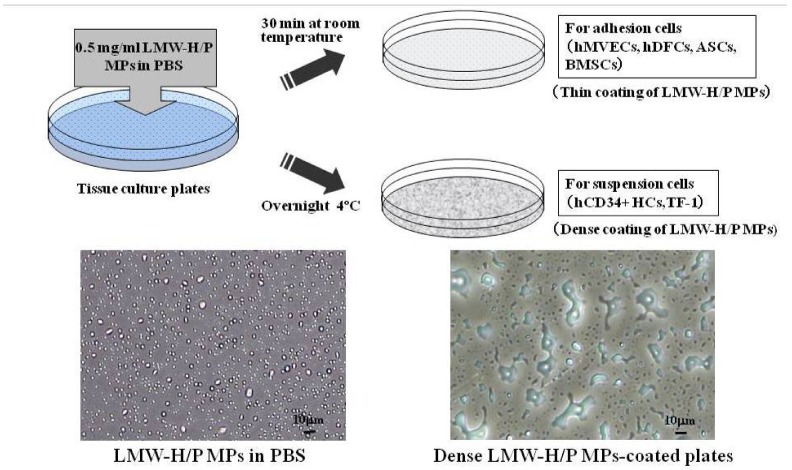
Preparation of F/P MPs-coated plates.

The LMW-H/P MPs are able to attach to polymeric surfaces such as plastic and glass. The LMW-H/P MPs generate a stable paste-like coating through complete drying. It is probable that polypeptides, such as FGF-2, interleukin (IL)-3 and granulocyte/macrophage-colony stimulating factor (GM-CSF), once bound to the LMW-H/P MPs-coated plates, are gradually released from the coated surface *in vitro* with a half-life of 4–6 days [26]. Furthermore, LMW-H/P MPs-coating could optimally stimulate growth of hMVECs and hDFCs in low FBS (1%)-DMEM with FGF-2 and growth of hematopoietic cell line (TF-1) with IL-3 and GM-CSF (Figure 7) [26].

LMW-H/P MPs generate a stable paste-like coating through complete drying. It is probable that polypeptides, such as heparin-binding cytokines, once bound to the LMW-H/P MPs-coated plates, are gradually released from the coated surface *in vitro*. Furthermore, LMW-H/P MPs-coating could optimally stimulate growth of hMVECs and hDFCs in low FBS-DMEM with FGF-2 and growth of hematopoietic cell line (TF-1) with adequate cytokines.

Heparin and heparinoids bind various GFs and cytokines including FGFs, HGF, VEGF, heparin-binding epidermal growth factor (HBEGF), PDGF, TGF-β, GM-CSF, interleukins (*i.e.*, IL-1, IL-2, IL-3, IL-4, IL-6, IL-7 and IL-8), interferon γ, and macrophage inflammatory protein-1 [41,42,43]. These GFs and cytokines can potentially be immobilized on the LMW-H/P MPs-coated plates. Actually, in addition to FGF-2, IL-3, and GM-CSF presented in this paper, we have already reported that FGF-1, HGF, HBEGF, TGF-β, human stem cell factor (SCF), thrombopoietin (Tpo), and Flt-3 ligand (Flt-3) could be efficiently immobilized on the LMW-H/P MPs-coated plates [29]. Thus, LMW-H/P MPs-coating provides an excellent biomaterial to immobilize and retain GFs and cytokines for optimal growth of various types of cells with low serum medium.

### 4.2. Culture Expansion of BMSCs and ASCs on LMW-H/P MPs-Coated Plates

Cell-based therapies such as tissue engineering will benefit from a source of autologous multipotent stem cells, including BMSCs or ASCs. There are two stem cell lineages in bone marrow cell populations, *i.e.*, hematopoietic cells (HCs) and BMSCs. The BMSCs and ASCs are multipotential, indicating that in culture [44,45] or after *in vivo* implantation these cells can differentiate into a variety of cell types including osteoblasts, chondrocytes, adipocytes, myoblasts [46], and neuronal cells [47]. 

Most protocols *in vitro* for the expansion of BMSCs or ASCs include high concentrations (10%–20%) of animal serum such as FBS as a nutritional supplement. In some cell cultures, this involves multiple doses of FBS, which raises concerns over possible contamination as well as immunological reactions caused by medium-derived FBS proteins, sialic acid derivatives, etc. [48,49]. Patients may experience problems when undergoing autologous cell-based therapies if a serum other than an autologous serum is used during the culturing of the cells. On the other hand, it would be difficult to obtain large amounts of autologous serum from the patient for large-scale autologous cell culture [27,28]. It should be noted that the growth of cultured BMSCs or ASCs on LMW-H/P MPs-coated plates in low FBS (1–2%) medium with FGF-2 was significantly stimulated, and similar stimulation was observed in those cultured cells on LMW-H/P MPs-coated plates with FGF-2 and 1–2% human serum (HS) prepared from adult bloods instead of FBS [27,28]. Thus, LMW-H/P MPs may serve as an effective matrix for cultures of BMSCs or ASCs. The safe and effective expansions of BMSCs or ASCs represent a promising option for tissue engineering strategies.

### 4.3. Proliferation of CD34+ Hematopoietic Progenitor Cells (CD34+ HCs) on LMW-H/P MPs-Coated Plates

Hematopoietic progenitor cells proliferate and mature in semi-solid media when stimulated by exogenous hematopoietic cell growth factors (HCGFs) such as SCF, Tpo, Flt-3, IL-3, and GM-CSF [27,29,50,51]. These cells also proliferate in association with bone marrow-derived stromal cells (BMSCs) [28,52,53], although biologically active amounts of HCGFs cannot be detected in stromal culture supernatants [53]. It is possible that HCGFs are synthesized by the stromal cells but remain bound to the stromal cells and/or their extracellular matrix. In fact, it was demonstrated that both natural and recombinant HCGFs, such as IL-3 and GM-CSF, could be adsorbed by heparan sulfate, which is the major sulfated glycosaminoglycan of bone marrow stroma [28,52,53]; however, it was reported that serum-free media require large amounts of SCF, Tpo, and Flt-3 to proliferate CD34+ HCs [29,54,55]. Although such media are commercially available (HPGM, Lonza Japan Corp. Tokyo, Japan), they are prohibitively expensive. In our previous study, we demonstrated that recombinant HCGFs such as SCF, Tpo, and Flt-3 were immobilized onto LMW-H/P MPs-coated plates, and the immobilized cytokines were gradually released into the medium. Furthermore, these cytokines, once bound, can be presented in the biologically active form to hematopoietic progenitor cells [29]. Furthermore, only one-fourth of the concentration of the cytokines recommended by the manufacture was required for maximal expansion of CD34+ HCs on the LMW-H/P MPs-coated plates [29]. These findings may have important implications for the use of heparinoid as an artificial matrix for *ex vivo* expansion of hematopoietic progenitor cells with adequate cytokines. The LMW-H/P MPs-coating matrix in the presence of lower concentrations of SCF, Tpo, and Flt-3 is a convenient and safe material for stable expansion of CD34+ HCs using HPGM without any animal serum. 

## 5. Conclusions

It is recognized in polymer chemistry that positively and negatively charged polymers interact ionically [21]. Through these ionic interactions, basic protamine molecules can bind with acidic molecules (LMW-H: fragmin) to form microparticle complexes. We previously reported that GFs-containing LMW-H/P MPs, which are about 0.5–3 μm in diameter, can be easily injected [22,23]. Furthermore, the effects of FGF-2- or PRP-containing LMW-H/P MPs were observed in the protection from heat and proteolytic inactivation of FGF-2 or GFs in PRP activity. These results indicate that LMW-H/P MPs may serve as an effective microcarrier for various GFs, particularly for the local application of GFs. GFs containing LMW-H/P MPs show a substantial effect to induce vascularization and fibrous tissue formation because of the gradual controlled release, protection and activation of GF molecules from GFs-containing LMW-H/P MPs [22,23,24] (Figure 3). 

LMW-H/P MPs rapidly bound to adhesive cell surfaces such as ASCs through specific interactions of LMW-H/P MPs and various cell surface heparin-binding proteins, can promote cell-to-cell interaction and increase cellular aggregation. The cells/LMW-H/P MPs-aggregate formation substantially promoted cell viability *in vitro*. The ASCs/LMW-H/P MPs-aggregates induced vascularization and fibrous tissue formation *in vivo* [25]. The LMW-H/P MPs, in combination with ASCs, are a new convenient cell carrier and may be a promising novel therapy for inducing vascularization and fibrous tissue formation in ischemic disease (Figure 5). LMW-H/P MPs also bind to the surface of various adhesive tumor cells, promoting cell-to-cell interaction and increase cellular aggregation with the LMW-H/P MPs. The tumor cells/LMW-H/P MPs-aggregates substantially promote cell survival and proliferation of those tumor cells *in vitro*, and reliably induce tumor formation and rapid tumor growth *in vivo* [26]. Taken together, LMW-H/P MPs constitute a new convenient and effective biomaterial that function as a tumor cell carrier *in vivo*. The application of LMW-H/P MPs as tumor cell carrier offers a more reliable model in both allograft and xenograft transplantation for cancer research. 

The presented method for the optimal proliferation and differentiation of ASCs and BMSCs on LMW-H/P MPs-coated plates in low concentration human serum medium (1–2%) is supplemented with FGF-2 (5 ng/mL). No animal serum is required in the culture of those cell types. The proliferated cells maintained their potential to differentiate into adipocytes and osteoblasts [25,28]. Furthermore, the LMW-H/P MPs-coating matrix in the presence of lower concentrations of SCF, Tpo, and Flt-3 were convenient materials for stable expansion of CD34+ HCs using HPGM without any animal serum [29]. These results suggest a promising cell source, particularly for the preparation of large amounts of ASCs, BMSCs, or CD34+ HCs required for cell-based therapies in several clinical fields.

LMW-H (fragmin), protamine, autologous PRP and several GFs and cytokines are already in clinical use. Since autologous ASCs, BMSCs, or CD34+ HCs are available, the clinical safety of LMW-H/P MPs as protein- and cell-carriers is possible. Furthermore, ASCs, BMSCs, or CD34+ HCs can be efficiently expanded as cell sources for regenerative medicines with the use of LMW-H/P MPs-coated plates as a matrix without the need for animal serum or feeder cells.
